# The respiratory microbiome in bronchial mucosa and secretions from severe IgE-mediated asthma patients

**DOI:** 10.1186/s12866-017-0933-6

**Published:** 2017-01-19

**Authors:** Laura Millares, Guadalupe Bermudo, Vicente Pérez-Brocal, Christian Domingo, Marian Garcia-Nuñez, Xavier Pomares, Andrés Moya, Eduard Monsó

**Affiliations:** 1 Fundació Parc Taulí, Parc Taulí 1, Edificio Santa Fe, planta baja, 08208 Sabadell, Barcelona Spain; 2 CIBER de Enfermedades Respiratorias, CIBERES, Madrid, Spain; 3 Universitat Autònoma de Barcelona, Esfera UAB, Barcelona, Spain; 4 Fundació Insitut d’Investigació Germans Trias i Pujol, Badalona, Spain; 5 Department of Respiratory Medicine, Hospital Universitari Parc Taulí, Sabadell, Spain; 6 Genomics and Health Area, Fundación para el Fomento de la Investigación Sanitaria y Biomédica de la Comunidad Valenciana (FISABIO-Public Health), Valencia, Spain; 7 CIBER Epidemiología y Salud Pública (CIBERESP), Madrid, Spain; 8 Evolutionary Genetics Unit, Institut Cavanilles de Biodiversitat i Biologia Evolutiva (ICBiBE), Universitat de València, Valencia, Spain; 9 Department of Medicine, Universitat Autònoma de Barcelona (UAB), Barcelona, Spain

**Keywords:** Microbiome, Asthma, Biopsy, Bronchial aspirate

## Abstract

**Background:**

The bronchial microbiome in chronic lung diseases presents an abnormal pattern, but its microbial composition and regional differences in severe asthma have not been sufficiently addressed. The aim of the study was to describe the bacterial community in bronchial mucosa and secretions of patients with severe chronic asthma chronically treated with corticosteroids in addition to usual care according to Global Initiative for Asthma. Bacterial community composition was obtained by 16S rRNA gene amplification and sequencing, and functional capabilities through PICRUSt.

**Results:**

Thirteen patients with severe asthma were included and provided 11 bronchial biopsies (BB) and 12 bronchial aspirates (BA) suitable for sequence analyses. Bacteroidetes, Firmicutes, Proteobacteria and Actinobacteria showed relative abundances (RAs) over 5% in BB, a cutoff that was reached by *Streptococcus* and *Prevotella* at genus level. *Legionella* genus attained a median RA of 2.7 (interquartile range 1.1–4.7) in BB samples. In BA a higher RA of Fusobacteria was found, when compared with BB [8.7 (5.9–11.4) vs 4.2 (0.8–7.5), *p* = 0.037], while the RA of Proteobacteria was lower in BA [4.3 (3.7–6.5) vs 17.1 (11.2–33.4), *p* = 0.005]. RA of the *Legionella* genus was also significantly lower in BA [0.004 (0.001–0.02) vs. 2.7 (1.1–4.7), *p* = 0.005]. Beta-diversity analysis confirmed the differences between the microbial communities in BA and BB (R^2^ = 0.20, *p* = 0.001, Adonis test), and functional analysis revealed also statistically significant differences between both types of sample on *Metabolism*, *Cellular processes*, *Human diseases*, *Organismal systems* and *Genetic information processing* pathways.

**Conclusions:**

The microbiota in the bronchial mucosa of severe asthma has a specific pattern that is not accurately represented in bronchial secretions, which must be considered a different niche of bacteria growth.

**Electronic supplementary material:**

The online version of this article (doi:10.1186/s12866-017-0933-6) contains supplementary material, which is available to authorized users.

## Background

The respiratory microbiome plays a role in the etiology and pathogenesis of lung diseases, and a thorough characterization of the microbial communities in relevant spatial niches of the respiratory system is needed for the understanding of their complex interactions [1–4]. Studies focusing on the respiratory microbiome in chronic respiratory diseases characterized by chronic airway inflammation, such as chronic obstructive pulmonary disease (COPD), cystic fibrosis (CF) and asthma have reported clear-cut differences in the composition of the microbial community with regard to healthy individuals, with an increased presence of members of the Proteobacteria phylum [5–7]. These changes are probably related to the structural and functional modifications of the respiratory mucosa which characterize these diseases and are modulated by the repeated corticosteroid and antibiotic treatments received by most of these patients [8–11].

The respiratory tract extends from the nasal and oropharyngeal cavities to the alveoli and includes niches with specific patterns in their microbiome composition [12, 13]. Microbiome studies of the respiratory system have been based until now on bronchoalveolar lavage (BAL), which samples the lower bronchi, and is considered representative of the bronchial mucosa [14], and sputum, a non-invasive proximal sample which comprises mainly bronchial secretions [14–17]. The composition of the respiratory microbiome in the upper and lower respiratory tree is very similar in the healthy subject [18], but spatial heterogeneity of the bacteria community within bronchi and lung has been reported in COPD and CF [2, 19], supporting the hypothesis that the respiratory system has significant regional differences in its microbial composition in patients with chronic respiratory diseases. In addition, bacteria often grow in biofilms, which are microbial sessile communities embedded in a matrix of extracellular polymeric substances produced by the bacteria that remain attached to host interfaces [20]. Bacteria in biofilm show an altered growth rate and gene transcription phenotype compared with the same microorganisms growing planktonically [20, 21] and are protected against host clearance mechanisms and antibiotic therapy [20]. Studies of the respiratory microbiome using culture-independent techniques in asthma have mainly described the microbial composition of the bacterial community recovered from sputum, but knowledge of the microbiome lodged in the bronchial mucosa and its functional characteristics is incomplete [7, 22, 23].

Most research on the respiratory microbiome has targeted chronic diseases other than asthma, and the bacterial communities living in the bronchial mucosa in severe asthma are largely unknown. Furthermore, a thorough analysis of different samples may provide a clear characterization of the bacterial communities living in different niches of the respiratory tract, which may include bacteria uncommonly found in bronchial secretions in the healthy subject, which may have an impact in the progression of the disease. The aim of this study was to describe the bacterial community lodged in the bronchial mucosa and in respiratory secretions, as well as their functional capabilities, in patients with severe chronic asthma receiving long-term oral corticosteroid treatment. Differences in the bacterial microbiota between biopsy samples obtained from a subsegmentary bronchus and simultaneously recovered bronchial aspirates, which represent bronchial secretions, were assessed in order to determine the regional distribution and functional patterns of the bronchial microbiome in this disease.

## Methods

### Design and population

This cross-sectional study was part of a project whose objective was the characterization of the bronchial tree in severe IgE-mediated chronic asthma, and included patients who were members of a cohort regularly attending an asthma outpatient clinic in a university hospital [24]. Patients were categorized as step 5 for therapy, according to the Global Initiative for Asthma (GINA, http://ginasthma.org/), and were receiving oral corticosteroids to control their disease in addition to the usual standard care. Monoclonal antibody therapy with omalizumab had not been used in the treatment of the patients at inclusion. The present study focused on the microbiological characteristics of the bronchial mucosa, and included the assessment of the bronchial microbiome in bronchial biopsies and aspirates obtained through bronchoscopy. Exclusion criteria included previously diagnosed bronchiectasis or cystic fibrosis, an exacerbation and/or a hospital admission due to any cause within the previous 3 months, and any other severe disease needing regular therapy. Participants were examined after a minimum stability period of 12 weeks without using antibiotics for any reason or any changes in their regular treatment.

### Sociodemographic and clinical measurements

Sociodemographic and clinical data were recorded at enrollment, and included smoking habits, respiratory symptoms, exacerbations in the previous year and treatments. All patients performed forced spirometry and reversibility testing according to standard techniques [25]. Forced vital capacity (FVC) and forced expiratory volume in the first second (FEV_1_) were measured with the same dry rolling seal spirometer (Sibelmed, Sibelgroup, Barcelona, Spain) and expressed as absolute values (mL) and percentages of the reference values obtained from age- and height-adjusted selected volunteers from the province of Barcelona [26].

### Bronchoscopy and sample collection

Bronchoscopy was performed using a flexible videobronchoscope (BF180; Olympus Optical Co., Tokyo, Japan). Local anesthesia and sedation for the procedure were achieved using topical lidocaine spray and intravenous midazolam respectively, in accordance with standard recommendations [27, 28]. The bronchoscope, after its standard disinfection procedure, was introduced transnasally and passed through the vocal cords without aspiration to avoid contamination of the collected sample by oropharyngeal bacteria [18]. The bronchial tree was examined and a bronchial biopsy (BB) was performed at subsegmentary level on a bronchus that was macroscopically normal on white light examination. A bronchial aspirate (BA) was obtained during the procedure with the tip of the bronchoscope placed at the distal trachea and main bronchi.

### DNA extraction

BB samples were diluted with PBS and centrifuged at 15,000 g for 15 min. The pellet was digested with proteinase K overnight at 56 °C. BA samples were diluted in a volume 4 times the weight of the sample with a 1/10 dithiothreitol dilution (Sputasol, Oxoid, Hampshire, United Kingdom), incubated at 37 °C for 15 min, and centrifuged at 15,000 g for 15 min. Then, BB and BA samples were lysed with the same in-house lysis buffer, which composition has been previously detailed [16] and consists of 100 U/mL of mutanolysin, 47,700 U/mL of lysozyme and 2 U/mL of lysostaphin dissolved in autoclave-sterilized MiliQ water and sterilized again in autoclave. After cell lysis, different extraction kits were chosen to purify the DNA from BB and BA samples to obtain the highest DNA yield from each type of sample. DNA from BB samples was purified with Qiamp MinElute Virus Spin Kit (Qiagen, Helden, Germany), according to manufacturer’s instructions, and DNA from BA was purified with DNA extraction Kit (Ambion, ThermoFisher, MA, USA), following the manufacturer’s instructions. DNA was stored at −80 °C for further analysis.

### PCR amplification and sequencing of 16S rRNA gene

16S was amplified following the 16S Metagenomic Sequencing Library Preparation Illumina protocol (Part # 15044223 Rev. A, Illumina, CA, USA). The gene-specific sequences used in this protocol target the 16S V3 and V4 region. Illumina adapter overhang nucleotide sequences were added to gene-specific sequences, and primers were selected following Klindworth et al. [29]. Using the standard IUPAC nucleotide nomenclature, the full length primer sequences used to follow the protocol targeting this region were: 16S Forward primer = 5′- tcgtcggcagcgtcagatgtgtataagagacagcctacgggnggcwgcag-3′ and reverse primer = 5′- gtctcgtgggctcggagatgtgtataagagacaggactachvgggtatctaatcc-3′.

Microbial Genomic DNA (5 ng/μl in 10 mM Tris pH 8.5) was used to initiate the protocol. PCR conditions were 5 min of initial denaturation at 94 °C followed by 25 cycles of denaturation (30 s at 94 °C), annealing (30 s at 52 °C) and elongation (1 min at 72 °C). After amplification, the products were visualized in 2% agarose gels. Extraction controls were PCR amplified in parallel with the samples, and, although no bands were detected in the gel electrophoresis, were sequenced together with the samples. After 16S amplification, the multiplexing step was performed using Nextera XT Index Kit (FC-131-1096, Illumina). One microliter of the PCR product was run on a Bioanalyzer DNA 1000 chip (Agilent, CA, USA) to verify the size, with an expected size of ~550 bp. After size verification, the libraries were sequenced using a 2 × 300 bp paired-end run (MiSeq Reagent kit v3 MS-102-3001, Illumina), on a MiSeq Sequencer according to manufacturer’s instructions (Illumina).

Quality assessment was performed by the use of the PRINSEQ-lite program [30] with the following parameters: min_length: 50, trim_qual_right: 20, trim_qual_type: mean, trim_qual_window: 20. R1 and R2 from Illumina sequencing were joined using fastq-join from ea-tools suite [31].

### Sequence analysis and microbiome accession number

The Quantitative Insights Into Microbial Ecology (QIIME) pipeline 1.9.0 [32] was used for sequence processing to obtain taxonomic information using the Greengenes 13_8 sequence database as reference and the RDP classifier 2.2. The open reference operational taxonomic unit (OTU) picking method was used with UCLUST and PyNAST version 1.2.2 as alignment method. Chimeric sequences were detected in QIIME with ChimeraSlayer and were removed from the OTU table and from the phylogenetic tree to perform downstream analyses.

In order to assess the influence of the reagent contamination in our samples, we sequenced 4 extraction controls and 1 PCR negative control. We obtained a mean of 633 (SD 469) sequences in these controls, which were processed in QIIME, the same way than the samples. Sixty-four different genera from 11 eleven phyla were identified in the negative controls, 37 of them with relative abundance >1% in at least one sample.

Following Bitiinger et al. [33] Fisher exact test was used to compare the overall frequency of occurrence of each genus between samples and controls. Genera showing in controls relative abundances (RAs) exceeding that in samples were considered as potential contaminants and were removed for subsequent analyses, and common contaminant genera were also checked in the samples and eliminated when present [34]. After removing all the contaminant OTUs from the final OTU table, downstream analyses were performed to determine alpha and beta-diversity.

Bacterial 16S rRNA data sets from this study are accessible in the European Nucleotide Archive under the study PRJEB12006 with the sample numbers ERS1014176-199.

### Functional analysis

The PICRUSt (Phylogenetic Investigation of Communities by Reconstruction of Unobserved States) software package [35] was used for the predictive functional analysis. This software estimates the community metagenome using 16S rRNA sequencing data. KEEG (Kyoto Encyclopedia of Genes and Genomes) pathway [36] was used to identify metagenomic contents. Abundance profiles of functional annotations were obtained and differences in the functional genomic content were evaluated after normalizing the abundances of each category to the total number of proteins predicted for each sample [37].

### Statistical analyses

Statistical analyses were performed using the SPSS statistical software package version 18 (SPSS Inc., Chicago, IL, USA). Results obtained from categorical variables are expressed as absolute and relative frequencies, and results for continuous variables as means and standard deviations (SD), when the distribution was normal or as medians and interquartile range (IQR) when the distribution was not normal. Bacterial α-diversity was assessed through the Chao1 estimator [38] and the Shannon index [39], calculating both indexes after subsampling with QIIME to avoid sequencing effort bias. Principal Coordinates Analysis (PCoA) with Bray-Curtis dissimilarity index [40] was used to study community composition, assessing the statistical significance of the differences in sample groupings through Adonis testing. Random forest analysis with Boruta feature selection was used to select the specific OTUs that were important in each type of sample using R package (http://www.r-project.org). Functional categories and their RA in both samples were compared using the Wilcoxon test. Statistical tests used in the study were two-sided, and a *p* value of 0.05 or less was reported as statistically significant.

## Results

### Patient characteristics

Thirteen patients with severe asthma were included in the study and provided 11 BB and 12 BA suitable for sequence analyses. Eight patients were women and five men with a mean age of 49 years (SD 14) and a FEV_1_ of 74% (SD 18.6). All had been receiving chronic treatment with oral corticosteroids for the control of their disease for a minimum period of 1 year.

### 16S rRNA analysis in BB samples

In BB samples, six phyla showed a RA over 1%: Bacteroidetes [median 34.1 (interquartile range (IQR) 20.2–37.3)], Firmicutes [27.9 (20.9–35.1)], Proteobacteria [19.1 (12.1–30.1)], Actinobacteria [7.8 (6.4–11.2)], Fusobacteria [4.3 (0.8–7.2)] and TM7 [1.1 (0.4–3.1)]. At genus level, this 1% cutoff was reached by 17 genera with *Streptococcus* [12.3 (2.3–15.7)] and *Prevotella* [11.4 (1.6–16.3)] being the most prevalent (Additional file 1: Table S1). *Legionella* and *Haemophilus* genera were found in all BB samples with median RA above 2% [2.7 (1.1–4.8) and 2.1 (0.6–4.1) respectively], while *Pseudomonas* genus was only found with median RAs below 1% [0.9 (0.3–1.9)] (Table 1).

**Table 1:** Genera with median relative abundances >1% detected in BB samples (*n* = 11)

Genera	Median (IQR)
*Streptococcus*	12.3 (2.3–15.7)
*Prevotella*	11.4 (1.6–16.3)
*Flavobacteriaceae*_g	4.5 (0.7–6.3)
*Legionella*	2.7 (1.1–4.7)
*Fusobacterium*	2.5 (0.8–4.9)
*Staphylococcus*	2.4 (0.5–3.7)
*Haemophilus*	2.1 (0.6–4.1)
[*Prevotella*]	2.1 (0.5–4.6)
*Cloacibacterium*	1.9 (0.3–4.4)
*Chryseobacterium*	1.9 (0.3–3.2)
*Gemellaceae*_g	1.9 (0.1–3.1)
*Legionellaceae*_g	1.7 (0.6–3.1)
*Acinetobacter*	1.7 (0.1–2.9)
*Porphyromonas*	1.5 (0.2–7.7)
*Enhydrobacter*	1.2 (0.2–2.4)
*Oxalobacteraceae*_g	1.1 (0.3–2.5)
*Corynebacterium*	1.1 (0.5–2.8)

### Comparison between bronchial aspirate (BA) and biopsy (BB) samples

In order to compare BA and BB 10 paired samples from the studied patients were used. The bacterial composition of BA samples showed statistically significant differences from BB at both phylum and genus levels. Bacteroidetes [36.1 (26.9–40.1)], Firmicutes [38.8 (32.3–44.2)], Proteobacteria [3.7 (3.7–6.5)], Actinobacteria [8.7 (6.9–12.6)], Fusobacteria [8.7 (5.9–11.4)], and TM7 [1.2 (0.2–3.1)] attained median RAs over 1% in BA, similarly found in BB. Fusobacteria, however, showed significantly higher RAs in BA [8.7 (5.9–11.4) vs *4*.2 (0.8–7.5), *p* = 0.037, Wilcoxon test], while the RA of Proteobacteria was significantly lower in BA when compared with BB [4.3 (3.7–6.5) vs 17.1 (11.2–33.4), *p* = 0.005, Wilcoxon test] (Table 2).

**Table 2:** Differences in the relative abundances of the phyla detected in BB and BA samples with median >1% at least in one type of sample

Phylum	BB (*n* = 10)	BA (*n* = 10)	
	Median (IQR)	Median (IQR)	*p* value
Bacteroidetes	34.4 (19.8–38.7)	36.1 (26.9–40.1)	0.799
Firmicutes	26.2 (20.2–36.4)	38.8 (32.2–44.1)	0.074
Proteobacteria	17.1 (11.2–33.4)	4.3 (3.7–6.5)	**0.005**
Actinobacteria	7.5 (6.3–11.3)	8.7 (6.9–12.6)	0.139
Fusobacteria	4.2 (0.8–7.5)	8.7 (5.9–11.4)	**0.037**
TM7	1.4 (0.3–3.1)	1.2 (0.2–3.1)	0.878

At genus level, higher RAs were found in BA for 28 genera, nine of them with median RAs over 1%, and among them *Prevotella* and *Streptococcus* attained levels over 20%. In BB, significantly higher RAs were found for 48 genera, with figures over 1% in 9 of them, including *Legionella* genus, which was only exceptionally found in BA. Overall, 76 out of 280 identified genera showed significant differences between BA and BB samples (Table 3).

**Table 3:** Genera with median relative abundances higher than 1% at least in one type of sample showing significant differences between BB and BA (higher abundances in bold types)

Genera	Biopsy (BB) (*n* = 10) Median (IQR)	Aspirate (BA) (*n* = 10) Median (IQR)	*p* value
*Streptococcus*	10.9 (2.2–15.8)	**24.2** (13.7–28.1)	0.007
*Prevotella*	12.2 (3.5–17.3)	**23.3** (12.1–26.9)	0.037
*Flavobacteriaceae*_g	**2.8** (0.6–5.7)	0.04 (0.007–0.08)	0.005
*Legionella*	**2.7** (1.1–4.7)	0.004 (0.001–0.02)	0.005
*Fusobacterium*	2.5 (0.8–5.7)	**4.9** (2.9–7.5)	0.047
*Staphylococcus*	**1.8** (0.4–3.5)	0.04 (0.02–0.3)	0.005
*Cloacibacterium*	**1.8** (0.3–3.7)	0 (0–0.0006)	0.005
*Legionellaceae_g*	**1.6** (0.5–2.7)	0.004 (0.0002–0.02)	0.005
*Chryseobacterium*	**1.3** (0.3–2.8)	0.01 (0.002–0.03)	0.005
*Gemellaceae*_g	1.5 (0.1–3.3)	**3.4** (2.3–4.6)	0.037
*Lactobacillus*	**1.2** (0.07–2.1)	0.02 (0.002–0.06)	0.005
*Corynebacterium*	**1.04** (0.5–2.8)	0.2 (0.1–0.4)	0.005
*Acinetobacter*	**1.03** (0.04–3.02)	0.005 (0.0008–0.01)	0.005
*Leptotrichia*	0.8 (0.2–2)	**3.3** (2.3–4.6)	0.005
*Rothia*	1.01 (0.2–2.2)	**3.1** (1.8–7.6)	0.007
*Actinomyces*	0.7 (0.4–1.3)	**2.7** (0.9–3.7)	0.017
*Atopobium*	0.6 (0.1–1.3)	**1.4** (0.8–2.3)	0.028
*Megasphaera*	0.8 (0.04–0.9)	**1.4** (0.8–2.6)	0.022

**Fig. 1:**
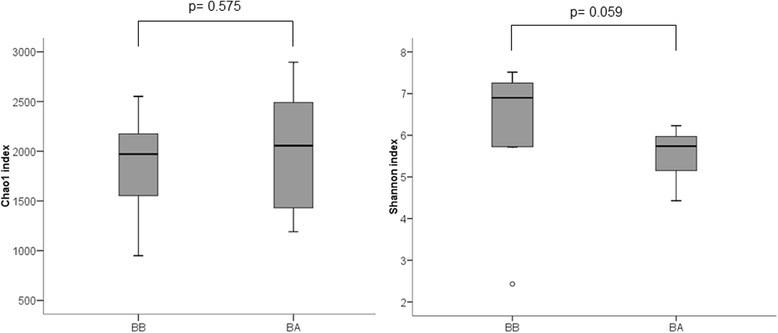
Chao1 index as a measure of richness and Shannon index as a measure of both richness and evenness in bronchial biopsy and aspirate

**Fig. 2:**
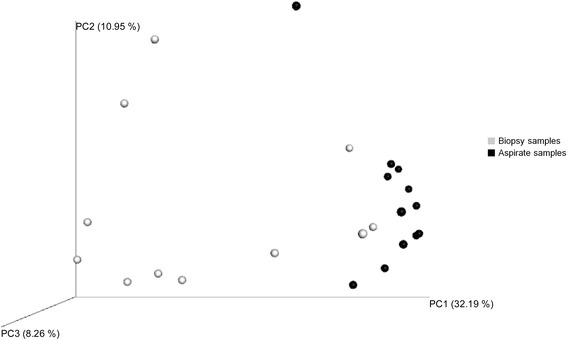
PCoA plot with Bray-Curtis β-diversity parameter. Biopsy samples in *grey* and aspirate samples in *black*

**Fig. 3:**
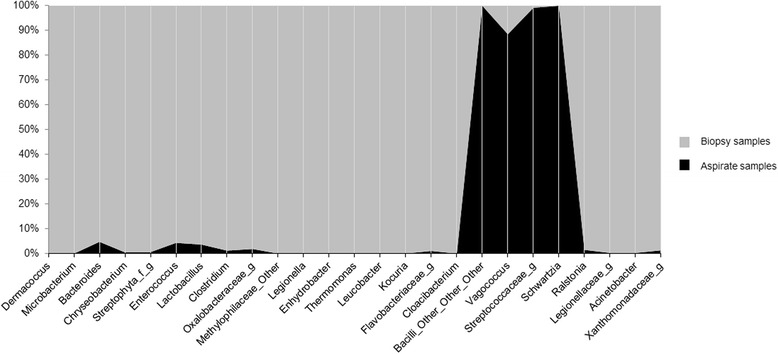
OTUs that discriminate between bronchial biopsy and aspirate, according to random forest analysis with boruta feature selection. Biopsy samples in *grey* and aspirate samples in *black*

Regarding bacterial α-diversity, Chao1 richness parameter did not show differences between BA and BB samples [2056.1 (1388.2–2517.2) vs. 1971.4 (1540.1–2217.1), *p* = 0.575, Wilcoxon test] and Shannon index, which combines both richness and evenness, was slightly lower in BA samples [5.7 (5.1–5.9) vs. 6.9 (5.7–7.3), *p* = 0.059, Wilcoxon test] (Fig. 1). This difference, although not statistically significant, suggests that the bacterial community had dominant taxa in BA. The assessment of the microbial composition using sample grouping at PCoA and the Bray-Curtis dissimilarity index showed also differences between BA and BB samples (Fig. 2), and Adonis testing confirmed that the microbial composition differed in both samples (R^2^ = 0.21, *p* = 0.001). Random forest analysis with Boruta feature selection identified 25 highly representative OTUs, four present only in BA samples and 21 with higher abundance in BB (Fig. 3). These OTUs were able to discriminate between BB and BA samples, and classified 11 out of 12 samples as BA and 10 out of 11 samples as BB.

### Functional analysis with PICRUSt

**Fig. 4:**
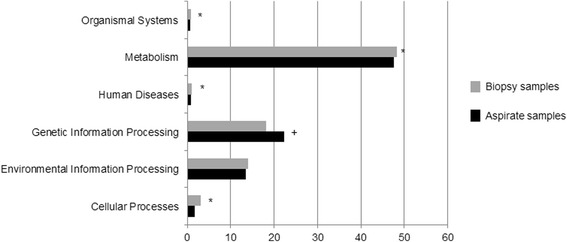
PICRUSt results at KEGG level 1. BB samples in *grey* and BA in *black*. Functional categories with significantly higher levels in BB (*) and BA (+) samples

**Fig. 5:**
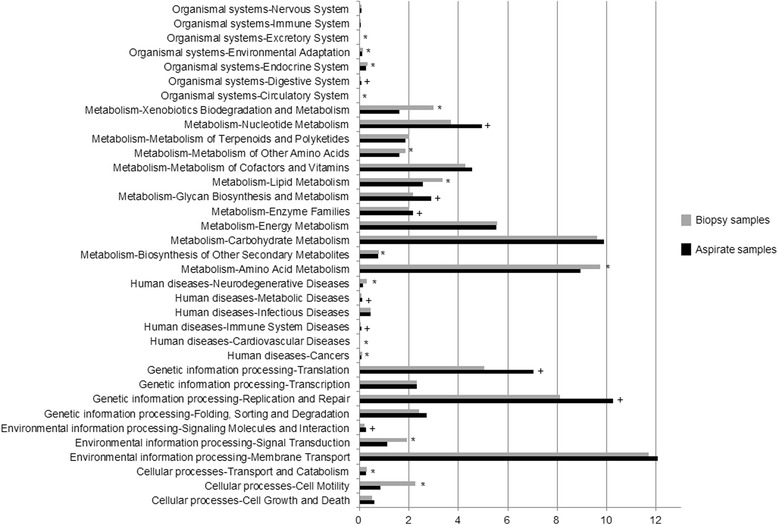
PICRUSt results at KEGG level 2. BB samples in *grey* and BA in *black*. Functional categories with significantly higher levels in BB (*) and BA (+) samples

The PICRUSt program was used to predict the functional capacities of the bacterial community from 16S rRNA sequences. From the six categories that make up KEGG level1, *Genetic information processing* was the only category which showed higher abundance in BA samples [22.3 (21.9–22.5) vs 18.1 (16.9–21.5), *p* = 0.017, Wilcoxon test], while *Metabolism*, *Cellular processes*, *Human diseases* and *Organismal systems* were significantly more abundant in BB [48.3 (47.9–48.9) vs 47.5 (46.9–47.7), *p* = 0.017; 3.1 (1.9–3.5) vs 1.7 (1.6–1.8), *p* = 0.009; 1.1 (0.9–1.1) vs 0.8 (0.8–0.9), *p* = 0.005 and 0.7 (0.6–0.8) vs 0.6 (0.6–0.6), *p* = 0.028 respectively] (Fig. 4). At KEGG level 2, 35 functional categories were detected, 24 with significant differences in their RA between BB and BA samples, nine higher in BA and 15 in BB (Fig. 5).

## Discussion

In this study we assessed the bacterial composition of the bronchial mucosa in severe chronic IgE-mediated asthma patients. Bacteroidetes, Firmicutes, Proteobacteria and Actinobacteria were the most abundant phyla, and *Prevotella* and *Streptococcus* the most predominant genera. Bacteria from *Legionella* genus were widely present in bronchial biopsies from severe asthma and attained RAs over 2% in most patients. Bronchial secretions showed a similar richness but clear-cut differences in their microbial composition, partly due to an overrepresentation of microorganisms from Bacteroidetes and Firmicutes phyla. These results confirm that the bronchial mucosa harbor a specific bacterial community in severe IgE-mediated chronic asthma, that is only partially represented by the microbiota of bronchial secretions.

In the present study, Bacteroidetes, Firmicutes and Proteobacteria phyla reached RAs over 15% in the bronchial mucosa of severe asthma patients, with *Streptococcus* and *Prevotella*, which attained RAs over 10%, as the most predominant genera. Proteobacteria phylum had significantly higher RA, a pattern that has been also reported for other severe chronic respiratory diseases such as COPD and CF [6, 8, 9], suggesting a common dysbiotic pattern in these obstructive diseases. The respiratory microbiome in healthy subjects is composed mainly of bacteria present in the oropharynx which migrate to the bronchial tree through aspiration and are found in the bronchial mucosa at low loads [13, 14, 41]. In subjects with chronic obstructive lung diseases such as COPD and CF, the structural and functional changes in the microenvironment conditions of the respiratory tract lead to a modification of their bacterial composition [41]. These changes favor an overgrowth of specific bacteria, mainly from the Proteobacteria phylum, which turn out to be well-adapted to the characteristics of the new environment, and is paralleled by a decline in the microbial diversity [17, 42]. This change is especially evident in patients with more severe disease, who show further increases in Proteobacteria and high levels of specific genera such as *Pseudomonas* [2, 8]. Despite the fact that our results confirm the increase of Proteobacteria abundance in bronchial mucosa, previously reported in asthma patients [6, 43], our patients did not show an overrepresentation of the genera *Haemophilus* and *Pseudomonas*, which is a frequent characteristic of severe COPD patients, and confirms that bronchial microbiome changes have specific patterns in severe asthma, also supported by the extensive identification of high loads of microorganisms from *Legionella* genus in the patients studied. These differences could be partially explained by tissue changes often observed in bronchial asthma and less frequent in COPD, such as an increase in tissue repair patterns [44].

Our results demonstrate that in asthma patients the microbiota recovered from bronchial secretions shows differences from the microbiota harbored in the bronchial mucosa. Firmicutes and Bacteroidetes had RAs over 25% in both BB and BA samples and were the most abundant phyla in both samples, but Proteobacteria phylum, which attained RAs over 15% in BB samples, was significantly less abundant in BA. At genus level, *Prevotella* and *Streptococcus* were the most abundant in both samples, although their proportions differed significantly in bronchial aspirates and biopsies. These two genera represented nearly a quarter of the RA of all the observed microbiota in BA samples, while in BB samples only accounted for slightly over 10% of the abundance. This finding is in agreement with α-diversity results, as shown by the Shannon index, which measures both richness and evenness, which was slightly lower in BA samples. Evenness is low in communities dominated by only a few species, and higher when the abundance is distributed equally among the wide range of species present in the sample. The differences observed in the bacterial communities of BA and BB samples were also found in the PCoA analysis performed. Studies of the bronchial microbiome of asthma patients have been based until now on bronchial brushings or bronchoalveolar lavage [6, 15, 43], which are considered representative of the bronchial mucosa on the basis of the results obtained in healthy subjects and COPD patients [14, 45], and in sputum samples [7, 22, 23]. The existence of substantial differences in the microbiota of bronchial secretions, sampled either through sputum or BA, and the bronchial mucosa confirms that these compartments should be considered to be different in patients with severe allergic chronic asthma, and supports the hypothesis that some of the microbial characteristics of the bronchial mucosa will be missed when only bronchial secretions are sampled in chronic respiratory diseases.

The presence in biopsy samples of biofilm-associated bacteria, such as *Pseudomonas* [21, 46, 47] and *Legionella* [48, 49], suggests that biofilm may be present on the bronchial mucosa of severe chronic asthma patients. Bacteria with the ability to form biofilms develop sessile communities which show major differences with respect to free-floating bacteria [21, 50]. BB appears to identify bacteria potentially embedded in biofilm and harbored in the bronchial mucosa, while BA samples seems to contain a major proportion of microorganisms with planktonic growth. Previous results of studies of the gastrointestinal tract have also shown that the microbiota of feces is not representative of the bacterial community of the gastrointestinal mucosa [51, 52]. The results obtained in respiratory samples from our study confirm that these differences in the bacterial composition between mucosa and luminal samples are not exclusive to the gut, and may be considered part of a pattern that includes the respiratory system.

The extensive finding of genus *Legionella* in the bronchial mucosa of severe allergic chronic asthma patients was unexpected. This genus was present in all BB samples where reached a median RA of 2.7%, and was significantly lower in BA samples. The identification of sequences with the Greengenes database was checked by aligning the sequences in BLAST and SINA [53], which confirmed the identification of all sequences as *Legionella*. To our knowledge, the presence of this genus in the bronchial mucosa of patients with chronic respiratory diseases such as asthma has not been previously reported, and future studies focusing on its role in patients following regular treatment with corticosteroids will be needed. *Legionella* species have been identified in non-malignant lung tissue obtained from lung cancer patients who underwent surgical resection [54], a finding in agreement with the hypothesis that *Legionella* may persist in human cells, against the common criteria that these microorganisms may be only found in patients with acute respiratory symptoms. *Legionella* is able to replicate intracellularlly in macrophages that have phagocytized aerosolized bacteria, and several studies have shown that *Legionella pneumophila* creates endoplasmic reticulum (ER)-like vacuoles that support intracellular replication. Through this mechanism the bacteria are able to interfere host autophagy processes, which is an ancient cell autonomous defense pathway based on ER-derived membranes to target intracellular pathogens for destruction [55–58]. The presence of *Legionella* in our BB samples could be explained by this mechanism, but future studies focused on the chronic presence of these bacteria in the respiratory system will be needed to confirm these findings and their medical implications.

Functional analysis using PICRUSt showed clear differences between the bacterial predicted metabolic functions in each type of sample in our study. *Cellular Processes*, *Metabolism*, *Organismal Systems* and *Human Diseases* pathways were significantly more abundant in bronchial tissue, while the *Genetic Information and Processing* pathway was more frequent in bronchial secretions. These findings seem also to be in accordance with previous studies showing that the gene expression of bacteria growing in biofilms differs from that of bacteria growing planktonically [20]. Biofilm bacteria have a specific growth rate and are more metabolically active [21], and the results obtained support the hypothesis of a wider metabolic activity of the microbiome found on the bronchial mucosa, identifiable through functional metagenomics.

The present study has some limitations that should be taken into account. First of all, the sample size was limited to 13 severe IgE-mediated chronic asthma patients; the inclusion of more subjects in future studies will be needed to confirm our results. Secondly, the patients enrolled had severe allergic asthma and were treated with oral corticosteroids, and the results obtained may not be extrapolable to patients with moderate asthma or patients who do not need chronic oral corticosteroid treatment. Thirdly, aspirate and biopsy samples are different approaches to sampling, and larger lung regions are sampled by aspirates. Therefore, the differences we obtained may be due to the method of sampling. Finally, we chose DNA purification methodologies to be used in every type of sample in order to obtain the maximum sample yield, a required approach for low biomass samples, as bronchial biopsies and aspirates, and we can not discard a marginal influence of the purification procedures followed on the obtained results.

## Conclusions

In severe chronic asthma there is high presence of Proteobacteria phylum in the bronchial mucosa, a microbial pattern that resembles COPD and CF, but shows specific findings as an extensive presence of *Legionella* genus, and low RAs of the genera *Haemophilus* and *Pseudomonas*. These findings confirm that the similarities between the bronchial microbiome of chronic obstructive respiratory diseases are only partial. Dominant taxa and lower evenness were observed in bronchial aspirates in these patients, confirming that bronchial secretions are only partially representative of the microbial communities from the bronchial mucosa, and confirm the existence of different niches for bacteria growth in the respiratory tree. Accordingly, the study underlines the inherent difficulties in obtaining a reliable description of the respiratory microbiome of the bronchial mucosa from the analysis of bronchial secretions.

## Additional file

Additional file 1: Table S1. Relative abundance (median, IQR) of all genera detected in BB and BA samples. (XLSX 32 kb)
